# Working memory and referential communication—multimodal aspects of interaction between children with sensorineural hearing impairment and normal hearing peers

**DOI:** 10.3389/fpsyg.2015.00242

**Published:** 2015-03-09

**Authors:** Olof Sandgren, Kristina Hansson, Birgitta Sahlén

**Affiliations:** Department of Logopedics, Phoniatrics, and Audiology, Clinical Sciences, Lund University, Lund, Sweden

**Keywords:** child hearing impairment, referential communication, working memory, phonological short term memory, gaze behavior

## Abstract

Whereas the language development of children with sensorineural hearing impairment (SNHI) has repeatedly been shown to differ from that of peers with normal hearing (NH), few studies have used an experimental approach to investigate the consequences on everyday communicative interaction. This mini review gives an overview of a range of studies on children with SNHI and NH exploring intra- and inter-individual cognitive and linguistic systems during communication. Over the last decade, our research group has studied the conversational strategies of Swedish speaking children and adolescents with SNHI and NH using referential communication, an experimental analog to problem-solving in the classroom. We have established verbal and non-verbal control and validation mechanisms, related to working memory capacity and phonological short term memory. We present main findings and future directions relevant for the field of cognitive hearing science and for the clinical and school-based management of children and adolescents with SNHI.

## LANGUAGE AND COMMUNICATION IN CHILDREN WITH SNHI

### LANGUAGE

The language development of children with sensorineural hearing impairment (SNHI) with hearing aids and/or cochlear implants has, at a group level, repeatedly been shown to depart from the typical trajectory. Several studies have found approximately half of preschool children with SNHI to exhibit substantial language problems, as compared to approximately 5% in the general population (Gilbertson and Kamhi, 1995; Hansson et al., 2004; Sahlén and Hansson, 2006), with particular deficits in phonological processing (Briscoe et al., 2001; Sahlén et al., 2004; Wake et al., 2006; Wass et al., 2008) and vocabulary (Mayne et al., 1998a,b; Hansson et al., 2004), whereas results are mixed regarding grammar (Norbury et al., 2001; Hansson et al., 2007). While basic language skills can normalize with age, children with SNHI have been found not to close the gap to normal hearing (NH) peers regarding complex language functioning, for example, oral and written narrative ability (Asker-Árnason et al., 2010; Reuterskiöld et al., 2010). Intrinsic (cognitive) and extrinsic (audiological and linguistic intervention, quality and quantity of input, feedback and teaching) factors, in complex interaction, likely contribute to the substantial heterogeneity in language outcome.

### COMMUNICATION

Whereas the primary purpose of language is communication, language ability—at least narrowly defined as the capacity to form linguistically coherent messages—is merely one tool necessary for successful communication. Verbal and non-verbal modalities are integrated with contextual factors to shape our ability to interact with others (Perkins, 2007). Interlocutors continuously merge the verbal message with information gathered from the partner’s speech, voice, posture, field of vision, gaze direction and gestures, as well as contextual information, for example, knowledge of the world, the context and the topic of the conversation. Consequently, intra- and inter-individual linguistic, cognitive, and socio-cognitive systems interact in communication. A hearing impairment may lead to misallocation of resources with negative effects on listening ability and understanding.

While studies often include protocols or checklists considered to capture social and communicative abilities there are surprisingly few experimental studies of children with SNHI interacting with others in everyday communicative settings. Most et al. (2010) analyzed aspects of pragmatic ability in 6 to 9 year-old children with severe-to-profound SNHI (using hearing aids and/or cochlear implants) from video recorded spontaneous conversation with a speech-language pathologist. Although not consistently impaired, the children with SNHI showed particular problems continuing the topic of the partner, and adding relevant information. Most et al. (2010) argued that the problems observed in the children with SNHI are caused by a delayed language development and limited linguistic input, resulting in an inexperience with various pragmatic behaviors and restricted perspective-taking. Compatible with a delayed language development, Toe and Paatsch (2010) presented results showing 7 to 12 year-old children with mild-to-profound SNHI to request repetition and clarification of questions to a significantly higher extent than NH peers. Similar results have been presented by our own research group using referential communication tasks, first introduced by Glucksberg and Krauss (1967), providing a compromise between experimental control and ecological validity, and designed to tap the communicative ability used in everyday activities such as giving instructions, describing things or events to a listener, and asking questions. In our studies, the referential communication tasks were designed to resemble communication between peers in structured classroom activities, rather than spontaneously occurring interaction.

### REFERENTIAL COMMUNICATION—METHODOLOGY

**FIGURE 1:**
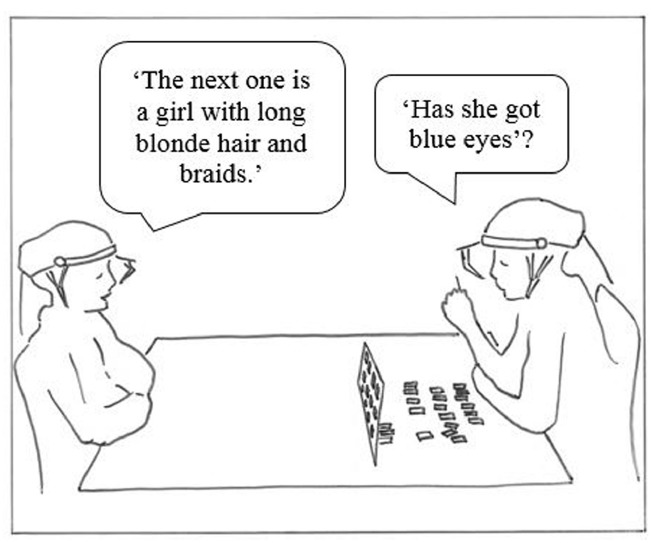
**Sketch of the experimental setting showing the speaker (on the left) describing pictures of faces, and the listener (on the right) requesting additional information.** Adapted from Sandgren et al. (2012).

Apart from providing details on typical communicative development, studies of referential communication have added to our knowledge of the communicative competence of individuals with a range of disabilities. In a referential communication task, the speaker is provided with an array of referents (pictures or physical objects), arranged in a predetermined pattern. The speaker’s task is to describe each picture/object, and its position, to enable the listener to arrange his/her array in the same way. Referential communication tasks allow investigation of the participants’ ability to produce (when in the “speaker” role), perceive and understand (when in the “listener” role) spoken messages (see Figure 1). Specifically, the task seeks to investigate whether the speaker can form contextually relevant messages, providing the listener with necessary information, without providing unnecessary details. The listener is evaluated on the ability to detect and resolve ambiguities through his/her use of questions. If, for example, the speaker describes a picture of a face as “It’s a man with a beard” this would provide sufficient information if all other referents lacked these characteristics. However, if the competing referents included other men with beards the listener would have to request additional information, for example “Is he wearing glasses?”

Referential communication requires a basic level of linguistic skills but also a range of cognitive capacities. The linguistic information must be processed and maintained until a referent has been chosen, requiring working memory capacity (WMC), the demands on which are likely to vary depending on the description provided (Dahlgren and Dahlgren Sandberg, 2008). Finally, in order for the speaker to provide an adequately detailed description, and for the listener to adjust his/her questions appropriately, both interlocutors must be able to take the perspective of the conversational partner.

### REFERENTIAL COMMUNICATION—FINDINGS

In a range of studies we have used an adapted version of the referential communication task, as a complement to linguistic and cognitive assessment, to investigate the communicative abilities of Swedish speaking children and adolescents with varying degrees of SNHI. While conducting the experiments under optimal acoustic conditions, with rigid experimental control, participants were instructed to choose a friend with whom to complete the task, thereby maintaining ecological validity. In the first study, Ibertsson et al. (2009a) found 11 to 19 year-old adolescents with severe-to-profound SNHI and cochlear implants to request more information than NH peers to resolve ambiguities caused by inaccurate or insufficient information from the conversational partner. The participants showed an increased use of requests for confirmation (yes/no questions, for example, “Does she have blonde hair?”), as compared to requests for elaboration (“What color is her hair?”). This use of questions was interpreted as a conversational strategy aimed at limiting the number of possible responses from the partner and thereby reducing the risk of misunderstanding. This conversational strategy was found to be related to complex WMC (Ibertsson et al., 2009b). Participants with SNHI and reduced WMC were found to use requests for confirmation of information mentioned earlier in the conversation (“Did you say he had a beard?”) whereas participants with greater WMC requested confirmation of new information to a greater extent, more clearly driving the conversation forward (Ibertsson et al., 2009b). Responses to the requests have not been shown to differ between the groups (Sandgren et al., 2011).

In an effort to obtain a fuller picture of the communicative exchanges during referential communication—both speech and body communication—we recently fitted interlocutors with mobile eye trackers (Sandgren et al., 2012, 2013, 2014). We were able to show that moments of mutual gaze, in which the listener looks at the speaker, showed a tight temporal connection with important parts of the spoken message (Sandgren et al., 2012). Questions, back-channeling responses, and statements, directed from the listener to the speaker, were all associated with higher probability of listener gaze to the speaker’s face. The results indicate that the spoken message is emphasized by the gaze exchanges, even to the point of making the content of the spoken message relevant. In a recurring example from the data, questions remained unanswered when not accompanied by a gaze to the respondent’s face (Sandgren et al., 2012). In a comparison between 10 and 15 year-old children with mild-to-moderate SNHI (mean age 12;6, SD 2;0; mean better ear pure-tone average 33.0 dB HL, SD 7.8) and NH same-age peers, the gaze behavior was found to be accentuated in the participants with SNHI, showing greater odds (ORs 1.2–2.1) for gaze to the speaker’s face than NH peers (Sandgren et al., 2014).

Since other factors than hearing differ between children with and without hearing impairment, we went on to investigate group differences in the probability of gaze to the speaker’s face while adjusting for individual performance on receptive grammar, expressive vocabulary, complex WMC, and phonological short term memory (PSTM; Sandgren et al., 2013). In the collected sample (cf. Sandgren et al., 2014 above), children with SNHI performed significantly below NH controls on non-word repetition (measuring phonological processing and PSTM) and expressive vocabulary, while non-significant differences were found for receptive grammar and complex WMC.

The group difference in gaze behavior remained significant despite adjustment for receptive grammar, expressive vocabulary, and complex WMC, but not non-word repetition, revealing an interaction between SNHI and PSTM capacity. Participants with SNHI with lower scores on non-word repetition (>1.25 SD below NH mean) showed a twofold increase in the probability of gaze to the speaker’s face, whereas those with higher scores had a reduced probability of looking at the conversational partner (Sandgren et al., 2013).

## CONCLUSIONS AND IMPLICATIONS

To summarize, request strategies and gaze behavior in children with SNHI during referential communication represent control and validation mechanisms which go above and beyond what is explained by the hearing impairment alone, and the results highlight WMC and PSTM capacity as driving forces behind the effect. While active and competent conversational partners, the participants with SNHI exhibit conversational strategies distinct from those of NH peers despite optimal conditions (clear task objectives, known conversational partner, no time limit, and silent surroundings). The findings affect clinical and school-based management of hearing impairment as well as our theoretical assumptions of the course of development of hearing impairment and its consequences. Speech-language pathologists, audiologists, psychologists and teachers working with children with SNHI should be aware of an increased likelihood of language deficits, which require intervention and adaptations to ensure academic attainment. This is equally relevant for younger school-aged children, whose language deficits may be easy to detect, and for later school years, when language profiles may have changed and previously sufficient coping strategies are challenged as school demands increase and learning is expected in adverse listening conditions (Bishop, 2014). Relevant for all is a comprehensive and continual evaluation of communicative functioning, including formal assessment of language, cognition, and interaction.

Our findings support the notion of WMC and PSTM playing important roles in the integration of auditory and visual information during speech production and perception. As suggested by the Ease of Language Understanding model (Rönnberg et al., 2013), a mismatch between input and long term memory representations will evoke extrinsic processing of the acoustic signal, requiring cognitive effort and strategic use of multimodal information, in this case possibly increased use of questions and gaze behavior during conversation. Future studies should evaluate individual variability in these memory capacities in relation to contextual multimodal challenge and support in the search for an explanation for the heterogeneity in language and communication outcome for children with SNHI. This should also provide an answer to whether the changes in request strategies and gaze behavior are, indeed, compensatory. The need for thorough and systematic studies of communication in children with SNHI should, however, not preclude prompt implementation of effective interventions based on current theories of language learning in typical and atypical populations.

### Conflict of Interest Statement

The authors declare that the research was conducted in the absence of any commercial or financial relationships that could be construed as a potential conflict of interest.

## References

[B1] Asker-Árnason L. Ibertsson T. Wass M. Wengelin Å. Sahlén B. (2010). Picture-elicited written narratives, process and product, in 18 children with cochlear implants. Commun. Disord. Q. 31, 195–212 10.1177/1525740109337734

[B2] Bishop D. V. M. (2014). Ten questions about terminology for children with unexplained language problems. Int. J. Lang. Commun. Disord. 49, 381–415. 10.1111/1460-6984.12101 25142090 PMC4314704

[B3] Briscoe J. Bishop D. V. M. Norbury C. F. (2001). Phonological processing, language, and literacy: a comparison of children with mild-to-moderate sensorineural hearing loss and those with specific language impairment. J. Child Psychol. Psychiatry 42, 329. 10.1111/1469-7610.00726 11321202

[B4] Dahlgren S. Dahlgren Sandberg A. (2008). Referential communication in children with autism spectrum disorder. Autism 12, 335–348. 10.1177/1362361308091648 18579643

[B5] Gilbertson M. Kamhi A. G. (1995). Novel word learning in children with hearing impairment. J. Speech Hear. Res. 38, 630–642. 10.1044/jshr.3803.630 7674656

[B6] Glucksberg S. Krauss R. M. (1967). What do people say after they have learned to talk? Studies of the development of referential communication. Merrill Palmer Q. Behav. Dev. 13, 309–316.

[B7] Hansson K. Forsberg J. Löfqvist A. Mäki-Torkko E. Sahlén B. (2004). Working memory and novel word learning in children with hearing impairment and children with specific language impairment. Int. J. Lang. Commun. Disord. 39, 401–422. 10.1080/13682820410001669887 15204448

[B8] Hansson K. Sahlén B. Mäki-Torkko E. (2007). Can a ‘single hit’ cause limitations in language development? A comparative study of Swedish children with hearing impairment and children with specific language impairment. Int. J. Lang. Commun. Disord. 42, 307–323. 10.1080/13682820600933526 17514544

[B9] Ibertsson T. Hansson K. Mäki-Torkko E. Willstedt-Svensson U. Sahlén B. (2009a). Deaf teenagers with cochlear implants in conversation with hearing peers. Int. J. Lang. Commun. Disord. 44, 319–337. 10.1080/13682820802052067 18821114

[B10] Ibertsson T. Hansson K. Asker-Arnason L. Sahlén B. (2009b). Speech recognition, working memory and conversation in children with cochlear implants. Deafness Educ. Int. 11, 132–151 10.1179/146431509790559615

[B11] Mayne A. M. Yoshinaga-Itano C. Sedey A. L. (1998a). Receptive vocabulary development of infants and toddlers who are deaf or hard of hearing. Volta Rev. 100, 29–52.

[B12] Mayne A. M. Yoshinaga-Itano C. Sedey A. L. Carey A. (1998b). Expressive vocabulary development of infants and toddlers who are deaf or hard of hearing. Volta Rev. 100, 1–28.

[B13] Most T. Shina-August E. Meilijson S. (2010). Pragmatic abilities of children with hearing loss using cochlear implants or hearing AIDS compared to hearing children. J. Deaf Stud. Deaf Educ. 15, 422–437. 10.1093/deafed/enq032 20624757

[B14] Norbury C. F. Bishop D. V. M. Briscoe J. (2001). Production of English finite verb morphology: a comparison of SLI and mild-moderate hearing impairment. J. Speech Lang. Hear. Res. 44, 165–178 10.1044/1092-4388(2001/015) 11218100

[B15] Perkins M. (2007). Pragmatic Impairment. Cambridge: Cambridge University Press.

[B16] Reuterskiöld C. Ibertsson T. Sahlén B. (2010). Venturing beyond the sentence level: narrative skills in children with hearing loss. Volta Rev. 110, 389–406.

[B17] Rönnberg J. Lunner T. Zekveld A. Sörqvist P. Danielsson H. Lyxell B. (2013). The Ease of Language Understanding (ELU) model: theoretical, empirical, and clinical advances. Front. Syst. Neurosci. 7:31. 10.3389/fnsys.2013.00031 23874273 PMC3710434

[B18] Sahlén B. Hansson K. (2006). Novel word learning and its relation to working memory and language in children with mild-to-moderate hearing impairment and children with specific language impairment. J. Multilin. Commun. Disord. 4, 95–107 10.1080/14769670600929360 15204448

[B19] Sahlén B. Hansson K. Ibertsson T. Reuterskiöld Wagner C. (2004). Reading in children of primary school age—a comparative study of children with hearing impairment and children with Specific Language Impairment. Acta Neuropsychol. 2, 393–407.

[B20] Sandgren O. Andersson R. Van De Weijer J. Hansson K. Sahlén B. (2012). Timing of gazes in child dialogues: a time-course analysis of requests and back channelling in referential communication. Int. J. Lang. Commun. Disord. 47, 373–383. 10.1111/j.1460-6984.2012.00151.x 22788224

[B21] Sandgren O. Andersson R. Van De Weijer J. Hansson K. Sahlén B. (2013). Impact of cognitive and linguistic ability on gaze behavior in children with hearing impairment. Front. Psychol. 4:856. 10.3389/fpsyg.2013.00856 24302915 PMC3831191

[B22] Sandgren O. Andersson R. Van De Weijer J. Hansson K. Sahlén B. (2014). Coordination of gaze and speech in communication between children with hearing impairment and normal-hearing peers. J. Speech Lang. Hear. Res. 57, 942–951. 10.1044/2013_JSLHR-L-12-0333 24167237

[B23] Sandgren O. Ibertsson T. Andersson R. Hansson K. Sahlén B. (2011). ‘You sometimes get more than you ask for’: responses in referential communication between children and adolescents with cochlear implant and hearing peers. Int. J. Lang. Commun. Disord. 46, 375–385. 10.3109/13682822.2010.507617 21771214

[B24] Toe D. M. Paatsch L. E. (2010). The communication skills used by deaf children and their hearing peers in a question-and-answer game context. J. Deaf Stud. Deaf Educ. 15, 228–241. 10.1093/deafed/enq006 20299450

[B25] Wake M. Tobin S. Cone-Wesson B. Dahl H.-H. Gillam L. Mccormick L. (2006). Slight/mild sensorineural hearing loss in children. Pediatrics 118, 1842–1851. 10.1542/peds.2005-3168 17079553

[B26] Wass M. Ibertsson T. Lyxell B. Sahlén B. Hällgren M. Larsby B. Mäki-Torkko E. (2008). Cognitive and linguistic skills in Swedish children with cochlear implants—measures of accuracy and latency as indicators of development. Scand. J. Psychol. 49, 559–576. 10.1111/j.1467-9450.2008.00680.x 18826424

